# Post-operative ctDNA monitoring in stage I colon cancer: A case report

**DOI:** 10.3389/fonc.2022.1074786

**Published:** 2022-12-14

**Authors:** Stephanie L. Alden, Harmeet Dhani, Charuta C. Palsuledesai, Shifra Krinshpun, Adham Jurdi, Eric Christenson, Ilene Browner, Samuel Rosner

**Affiliations:** ^1^ Johns Hopkins Hospital, Department of Medicine, Baltimore, MD, United States; ^2^ Natera, Inc., Austin, TX, United States; ^3^ Medical Oncology, Johns Hopkins Sidney Kimmel Comprehensive Cancer Center, Baltimore, MD, United States

**Keywords:** circulating tumor (ctDNA), molecular residual disease, stage I, recurrence, case report, colon cancer

## Abstract

Circulating tumor DNA (ctDNA) level monitoring after surgery for colon cancer has been studied in stage II and III colon cancer to risk-stratify patients for adjuvant therapy. However, there is less data regarding the role of this diagnostic tool in the management of stage I disease, where current recommended surveillance is limited to screening colonoscopy at one year. In this report, we describe the case of a 57-year-old man with stage I colon cancer who underwent complete resection with adequate lymph node surgical sampling, normal preoperative CEA and no evidence of metastatic disease on initial imaging. The patient elected to undergo serial ctDNA monitoring after surgery. Rising ctDNA levels, five months after resection, prompted cross-sectional imaging which demonstrated metastatic disease to the liver. The patient subsequently received five cycles of leucovorin, 5-fluorouracil, oxaliplatin, and irinotecan with bevacizumab (FOLFOXIRI-Bev) and definitive microwave ablation to the liver metastases, with resulting undetectable ctDNA levels. The patient’s imaging and colonoscopy one-year post-operatively showed no evidence of disease, with ctDNA levels remaining undetectable. This report highlights the value of ctDNA monitoring in patients with early-stage colon cancer and suggests that further, large-scale studies may be warranted to determine its appropriate clinical use.

## Introduction

While initial therapy for non-metastatic, resectable colon cancer is well-established, adjuvant chemotherapy is not recommended for those with stage I and low-risk stage II disease. Instead, surveillance of stage I disease after curative-intent resection solely involves colonoscopy one year after surgery (1). In stage II colon cancer, high risk factors such as degree of differentiation, pathological stage, lymphovascular invasion, obstruction or perforation, and/or inadequate lymph node sampling have guided clinicians to offer adjuvant chemotherapy, however, recent studies suggest that these criteria may not optimally select for patients who benefit from treatment, which may account for the approximately 3-8% and 12-24% three-year recurrence rate seen in patients with stage I and II colorectal cancer, respectively (2–5). Current staging guidelines that dictate surveillance strategies for early-stage disease use tumor size and depth of invasion (T), lymph node involvement (N), and metastatic disease (M) (6). This current staging system does not incorporate genomic markers of risk, although they play a major role in characterizing underlying disease biology, as seen in many other malignancies, such as breast cancer and renal cell carcinoma (6–9).

More recent data suggest that circulating tumor DNA (ctDNA) level is a useful biomarker in colorectal cancer to identify patients at high risk of recurrence, as well as which patients may benefit from adjuvant therapy (10, 11). Studies have demonstrated that patients with stage I disease have lower pre-operative ctDNA levels as compared to patients with stage II and III disease, consistent with a lesser overall disease burden (12). While patients with stage I colon cancer are less likely to recur than those with stage II or stage III disease, those who do recur post-operatively have detectable ctDNA levels before recurrence is detected *via* imaging (12, 13). However, most studies of ctDNA in assessing recurrence risk are limited to patients with stage II and stage III colon cancer, with stage I patients only accounting for 4-16% of study populations (12–14). Currently no randomized clinical trials have explored the use of ctDNA for disease monitoring and treatment stratification in stage I disease (15, 16).

In this clinical vignette, we present a patient with stage I colon cancer whose clinical course changed dramatically as a result of incorporating post-operative ctDNA testing into recommended surveillance, leading to earlier detection of metastasis, modification in treatment plan, and an overall more favorable outcome. Thus, this case highlights the potential impact of this diagnostic tool’s use in disease monitoring and perioperative management for early-stage colon cancer.

## Case description

**Figure 1:**
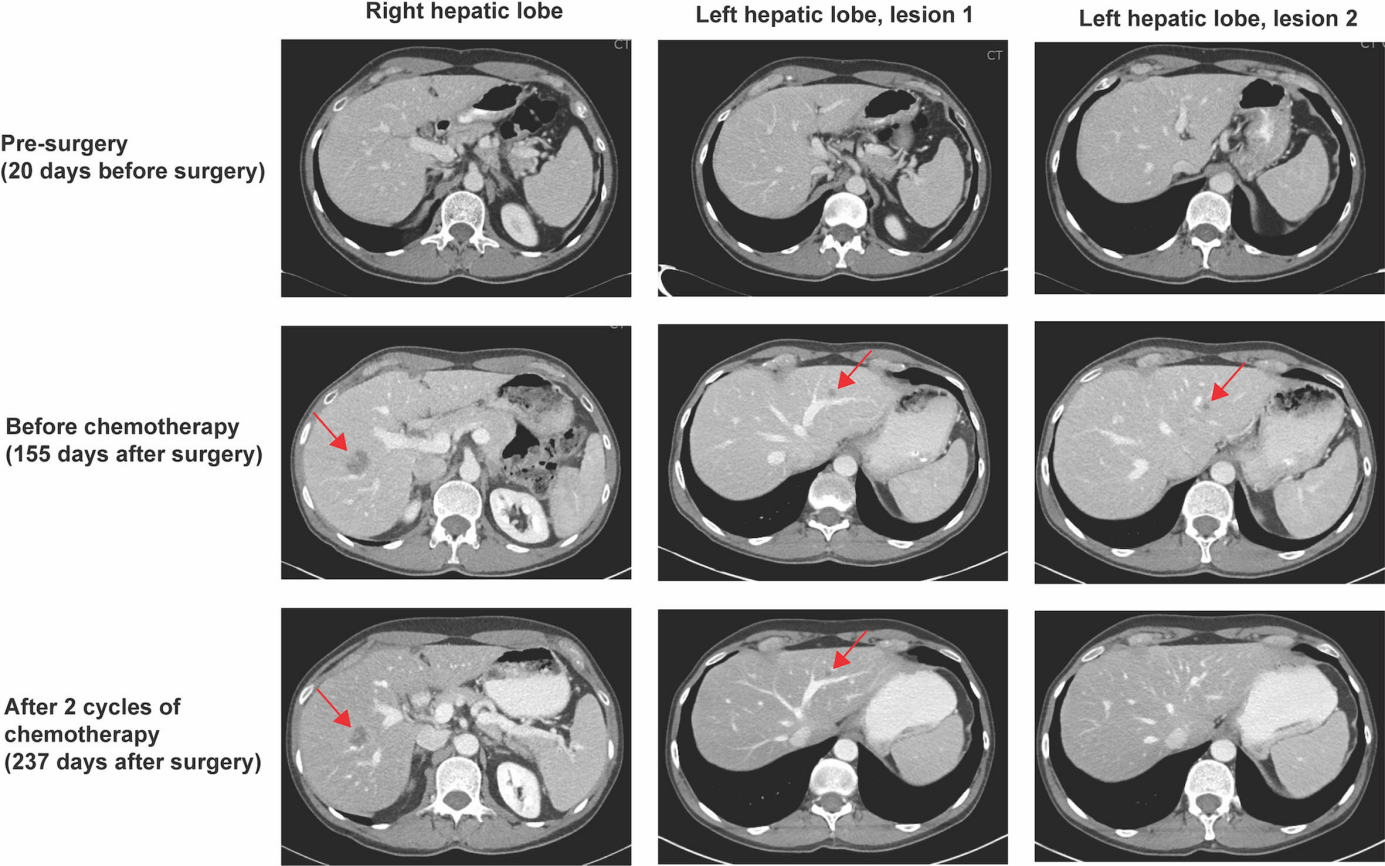
CT scan results. CT scan 20 days prior to right hemicolectomy did not show any evidence of metastatic disease (top panel). Rising ctDNA levels prompted a CT scan on post-operative day 155, which revealed a 2.4 x 1.8 cm ill-defined hypoattenuating lesion in the right hepatic lobe, as well as 0.8 cm and 0.4 cm nodules in the left hepatic lobe (red arrows, middle panel). An interval CT scan after two cycles of chemotherapy showed response of all hepatic lesions (red arrows, bottom panel).

**Figure 2:**
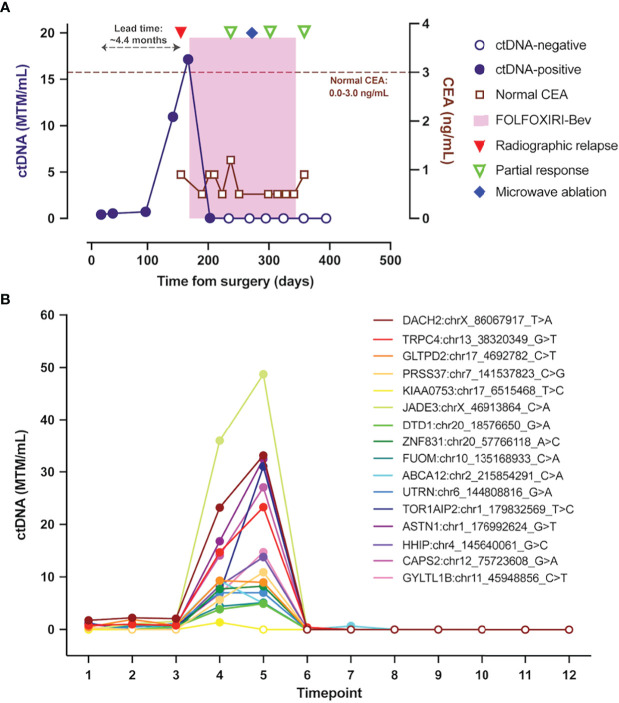
ctDNA dynamics throughout the clinical course. **(A)** Overview plot depicting composite ctDNA changes as measured in MTM/mL, along with radiographic imaging and therapeutic interventions. **(B)** Changes in levels of patient-specific variants identified and tracked using Signatera™ Molecular Residual Disease Test. ctDNA, circulating tumor DNA; MTM/mL, mean tumor molecules per milliliter of plasma.

A 57-year-old male patient presented with new onset rectal bleeding. Evaluation with colonoscopy showed a mass in the hepatic flexure, with biopsy demonstrating moderately differentiated adenocarcinoma. Initial fluorodeoxyglucose (FDG)-positron emission tomography (PET)/computed tomography (CT) demonstrated a small focus of FDG activity in the proximal transverse colon correlating with nodular soft tissue thickening on CT. No evidence of metastatic disease was present radiographically. An initial right hepatic lobe lesion noted on CT (**Figure 1**) and PET-scan was later confirmed to represent a benign hemangioma based on follow up magnetic resonance imaging (MRI). At the time of diagnosis, the patient had undetectable carcinoembryonic antigen (CEA) levels (**Figure 2A**).

The patient underwent a right hemicolectomy within one month of diagnosis. Histology showed a 2.1 cm lesion consistent with moderately differentiated adenocarcinoma with mucinous features invading the muscularis propria, with negative margins. Adequate lymph node sampling was performed, with 16 lymph nodes returning negative for adenocarcinoma. Final TNM staging was pT2N0M0, consistent with stage I colon cancer. Immunohistochemistry for mismatch repair (MMR) proteins demonstrated MMR-proficient tumor, with retained nuclear expression of MLH1, PMS2, MSH2, and MSH6. There was no lymphovascular invasion, perineural invasion, or macroscopic tumor perforation. Genomic analysis (Altera Tumor Genomic Profile, Natera, Inc.) showed that the tumor had low tumor mutational burden (TMB, 2 mutations/megabase), was microsatellite stable (MSS), and had genomic alterations including *APC* (D170 loss of function mutation), *APC* (E1397 stop gain mutation), *KRAS* (G12D), and PIK3R1 (K447_Y452 deletion).

Initially, the patient was offered surveillance with colonoscopy at one year after surgery, per National Comprehensive Cancer Network (NCCN) guidelines (1). However, after further discussion and shared decision-making, the patient pursued ctDNA testing using a personalized, tumor-informed platform (Signatera™ Molecular Residual Disease Test, Natera, Inc.). ctDNA was detected and quantified using a personalized, tumor-informed, multiplex PCR (mPCR) next-generation sequencing (NGS)-based assay (Signatera™, Natera, Inc.) as previously described (14). Briefly, a set of 16 high-ranked, patient-specific, somatic, clonal single nucleotide variants (SNVs) were selected for mPCR testing by whole-exome sequencing (WES) performed on formalin-fixed and paraffin-embedded (FFPE) tumor tissue and matched normal blood sample. The mPCR primers targeting the selected personalized SNVs were designed, synthesized, and used to track ctDNA in the patient’s longitudinal plasma sample. Plasma samples with at least two out of 16 SNVs detected were considered ctDNA-positive. ctDNA concentration was reported as mean tumor molecules (MTM) per mL of plasma.

The patient’s ctDNA level, measured using the assay described above, 25 days after surgery was 0.42 MTM/mL, with repeat ctDNA analysis six weeks after surgery indicating persistently low-level positive ctDNA at 0.55 MTM/mL (**Figure 2A**). Longitudinal ctDNA monitoring revealed an ongoing increase to 10.96 MTM/mL five months after surgery (**Figures 2A, B**). This rapid rise in ctDNA level prompted a CT scan of the chest, abdomen, and pelvis with contrast that detected a 2.4 x 1.8 cm ill-defined hypoattenuating lesion in the right hepatic lobe, as well as 0.8 cm and 0.4 cm nodules in the left hepatic lobe, concerning for oligometastatic disease (**Figure 1**). Given the patient’s baseline molecular and histopathologic profile, standard multi-drug chemotherapy regimens were discussed for management of his now oligometastatic disease (1). Taking into account the patient’s robust Eastern Cooperative Oncology Group (ECOG) performance status of 0, along with the goal of maximizing objective response to systemic therapy, a shared decision was made to initiate front-line fluorouracil, oxaliplatin, and irinotecan plus bevacizumab (FOLFOXIRI-Bev) to manage his oligometastatic colon cancer approximately five months after definitive surgery (1, 17).

Prolonged afebrile neutropenia requiring growth factor support complicated the patient’s initial chemotherapy course and led to a delay in cycle two. A ctDNA level after the first cycle of FOLFOXIRI-Bev decreased to 0.04 MTM/mL (**Figure 2A**). Interval imaging after two cycles of chemotherapy showed a response of all hepatic lesions, with ctDNA level cleared to undetectable (0.00 MTM/mL) (**Figure 1**; **Figure 2A**). After completing three cycles of chemotherapy, in consultation with surgical oncology, the patient elected to have definitive microwave ablation of the three hepatic metastases. The patient’s bevacizumab was held during the third cycle of chemotherapy, pre-operatively, but was resumed during his fourth cycle, post-ablation. The patient’s ctDNA level remained undetectable (0.00 MTM/mL) (**Figures 2A, B**) throughout the remainder of his adjuvant treatment course. Considering the lack of detectable ctDNA and persistent side effects from chemotherapy experienced by the patient, a shared decision was made to defer the sixth cycle of chemotherapy and, overall, the patient completed five cycles of adjuvant chemotherapy. Colonoscopy one year after surgery was unremarkable, and no new metastatic lesions were noted on repeat CT scan after chemotherapy completion. Of note, the patient’s CEA levels remained within normal limits (≤3.0 ng/mL per institutional assay) throughout his disease course, from diagnosis to recurrence to post-treatment monitoring (**Figure 2A**).

## Discussion

Current surveillance for early-stage colon cancer after definitive resection entails measuring CEA levels, routine blood work, CT imaging, and colonoscopy (1). However, these diagnostic methods frequently fail to capture patients with micrometastatic disease who will recur without additional intervention. This notion is particularly true for patients with stage I disease, where current guidance forgoes radiographic monitoring, and little data exists regarding the risk for recurrence (1–5). In the clinical case presented above, serial ctDNA measurement prompted additional imaging, leading to earlier intervention, with aggressive systemic therapy as a bridge to definitive treatment.

Our patient’s ctDNA levels after chemotherapy correlated with radiographic response after the first two cycles of chemotherapy. This combination of laboratory and radiologic improvement led the treatment team to pursue more definitive disease control with microwave ablation of his hepatic lesions. Consistent with findings in other studies, our patient’s CEA level was negative despite radiologic evidence of recurrence and positive ctDNA (**Figure 1**; **Figure 2A**), suggesting that the use of multiple biomarkers to monitor for recurrence may enable earlier disease detection (12, 14–16, 18, 19).

ctDNA monitoring after definitive treatment of stage II and stage III colon cancer has shown promise in assessing recurrence risk, with the major focus being the use of ctDNA level to guide escalation and de-escalation of therapy in the adjuvant setting (10, 11, 15, 16). These encouraging findings have raised enthusiasm over the potential role ctDNA may ultimately play in personalizing peri-operative treatment strategies based on dynamics of molecular response (15, 16). As a result, almost a dozen clinical trials have been completed or are underway to determine if post-operative and post-adjuvant chemotherapy ctDNA monitoring can be used to tailor treatment based on detection of post-operative ctDNA levels (15, 16, 20). Such efforts may help better select patients in need of more aggressive treatment approaches, while simultaneously sparing those with undetectable ctDNA levels who may not benefit from further chemotherapy (10, 15, 16).

While studies evaluating the use of ctDNA to guide treatment have focused on the potential to refine patient selection for adjuvant chemotherapy in stage II colon cancer and potentially de-escalate therapy in patients with stage III colon cancer, few studies have focused on the use of ctDNA to determine when to escalate therapy in stage I colon cancer (10, 11, 15, 16, 20). Our case suggests that ctDNA could be a useful tool to select patients who should undergo closer monitoring to detect early recurrence, with the result being curative treatment options. In those with stage I disease who do recur, ctDNA could also help guide duration and escalation of chemotherapy in the setting of recurrence. Anecdotally, in an interim analysis of the GALAXY trial, part of CIRCULATE Japan, the only patient with stage I colon cancer who was ctDNA positive after surgical resection eventually developed disease recurrence (21).

Given the anecdotal nature of a single report, findings and conclusions from this case may not be applicable to other clinical scenarios. Indeed, additional investigation into the accuracy and utility of ctDNA monitoring for early-stage disease is an ongoing area of research, albeit with significant clinical potential (22). The cost effectiveness of this intervention must also be taken into account, particularly given that patients with stage I disease are less likely to recur than those with stage II and III disease (16). Certainly, not all patients with lower risk stage I or II colon cancer require more intensive surveillance, however, non-invasive tools such as ctDNA monitoring may help further stratify patients to determine those who may benefit from additional monitoring and treatment. Looking forward, multi-modal approaches beyond the standard TNM staging classifications, with integration of genomic data, may better capture the risk of recurrence, as has been seen in other malignancies, such as lung adenocarcinoma (23). While still under investigation, such approaches may more accurately risk-stratify patients with early-stage disease.

## Conclusion

Our clinical case highlights the potential benefits of ctDNA monitoring in stage I colon cancer. This diagnostic tool can help identify patients at increased risk for recurrence. In our case, early detection of low-volume metastatic disease after ctDNA monitoring prompted early and aggressive treatment strategies, ultimately leading to a favorable clinical outcome.

## Data availability statement

The raw data supporting the conclusions of this article will be made available by the authors, without undue reservation.

## Ethics statement

Ethical review and approval was not required for the study on human participants in accordance with the local legislation and institutional requirements. The patients/participants provided their written informed consent to participate in this study. Written informed consent was obtained from the individual(s) for the publication of any potentially identifiable images or data included in this article.

## Author contributions

All authors contributed to the article and approved the submitted version.
